# Supplementary material for: A Likelihood Approach for Real-Time Calibration of Stochastic Compartmental Epidemic Models
Source: PLoS Comput Biol. 2017 Jan 17;13(1):e1005257. doi: 10.1371/journal.pcbi.1005257 (PMC5240920; doi:10.1371/journal.pcbi.1005257)
Supplement: S1 File — (TAR.GZ) [file pcbi.1005257.s014.tar.gz › HSPH_Online-SI-Revision/PF08/n100-severe/n100-severe_PF_table4.pdf]

1	R0	{1.65278, 9.06793}	{2.21433, 20.}	{{2.14624, 20.}, {2.27922, 20.}}	{33.9763, 120.558}	inf	4608.	15 575.5	{14 981.8, 16 175.7}	238.01	0.528685	newly	3152.	5101.58	{4900., 5327.}	61.8522	0.208861	Reff	0.666979	0.633228	{0.62153, 0.647023}	5.0604	ar	66 342.	96 646.2	{95 366., 97 921.}	45.6787
2	R0	{1.4321, 3.80644}	{3., 20.}	{{3., 20.}, {3., 20.}}	{109.483, 425.425}	inf	684.	17 995.2	{17 735.6, 18 181.9}	2530.88	1.42008	newly	952.	5604.87	{5496., 5715.}	488.747	0.769883	Reff	0.675178	0.335249	{0.330637, 0.340102}	50.3466	ar	54 217.	100 496.	{100 116., 100 824.}	85.3597
3	R0	{1.68388, 4.76028}	{3., 20.}	{{3., 20.}, {3., 20.}}	{78.16, 320.143}	inf	788.	19 601.3	{19 533.2, 19 657.7}	2387.47	1.39576	newly	834.	6105.23	{6007., 6207.}	632.042	0.864505	Reff	0.549854	0.347407	{0.34486, 0.349651}	36.8184	ar	68 299.	102 477.	{102 111., 102 818.}	50.042
4	R0	{1.66014, 6.95383}	{2.91617, 20.}	{{2.88247, 20.}, {2.96062, 20.}}	{75.6581, 187.611}	inf	3183.	17 804.5	{17 567.3, 18 100.7}	459.363	0.747663	newly	2735.	5577.1	{5457., 5710.}	103.916	0.309385	Reff	0.623814	0.376745	{0.365821, 0.389385}	39.6062	ar	66 820.	98 294.6	{97 693., 98 896.}	47.1036
5	R0	{1.69984, 11.2038}	{2.25736, 20.}	{{2.21603, 20.}, {2.29819, 20.}}	{32.7983, 78.5109}	inf	5824.	13 880.9	{13 582.2, 14 138.8}	138.339	0.377142	newly	3233.	4516.44	{4386., 4655.}	39.6981	0.145096	Reff	0.680072	0.600678	{0.576624, 0.623144}	11.6743	ar	68 462.	96 682.1	{95 773., 97 507.}	41.2201
6	R0	{1.65876, 6.66217}	{2.79683, 20.}	{{2.75854, 20.}, {2.82977, 20.}}	{68.6095, 200.202}	inf	3387.	19 428.7	{19 135., 19 781.8}	473.624	0.758598	newly	2924.	6159.98	{6030., 6307.}	110.67	0.323539	Reff	0.632386	0.452639	{0.439352, 0.463802}	28.4236	ar	67 105.	96 972.5	{96 131., 97 705.}	44.5087
7	R0	{1.69966, 5.89996}	{3., 20.}	{{3., 20.}, {3., 20.}}	{76.5059, 238.985}	inf	2191.	19 642.4	{19 443.7, 19 880.}	796.506	0.952539	newly	2014.	6142.65	{6028., 6249.}	204.998	0.484256	Reff	0.564848	0.367882	{0.36279, 0.372403}	34.8706	ar	69 396.	99 507.4	{99 241., 99 810.}	43.3906
8	R0	{1.53428, 6.3734}	{2.20078, 17.8285}	{{2.18716, 17.5296}, {2.21178, 18.0983}}	{43.4409, 179.733}	inf	2878.	15 425.8	{15 068.7, 15 708.1}	435.991	0.729083	newly	2677.	5592.64	{5496., 5676.}	108.914	0.31993	Reff	0.700629	0.611223	{0.605959, 0.614258}	12.7609	ar	59 855.	91 156.9	{90 329., 91 861.}	52.2963
9	R0	{1.58939, 3.48608}	{3., 20.}	{{3., 20.}, {3., 20.}}	{88.7517, 473.71}	inf	666.	25 798.9	{25 753.6, 25 832.4}	3773.7	1.58813	newly	876.	8267.57	{8155., 8363.}	843.787	0.974853	Reff	0.606066	0.529159	{0.527538, 0.530855}	12.6895	ar	62 575.	97 889.4	{97 717., 98 043.}	56.4353
10	R0	{1.58528, 5.5593}	{1.97388, 20.}	{{1.97298, 20.}, {1.97728, 20.}}	{24.5133, 259.758}	inf	2018.	15 541.	{15 531.7, 15 580.5}	670.117	0.886554	newly	2025.	5365.55	{5285., 5464.}	164.965	0.423151	Reff	0.636823	0.947453	{0.946246, 0.948491}	48.7782	ar	63 039.	84 446.9	{84 116., 84 710.}	33.9597
11	R0	{1.58305, 4.62721}	{3., 20.}	{{3., 20.}, {3., 20.}}	{89.5076, 332.226}	inf	1344.	19 196.5	{19 011.7, 19 504.}	1328.31	1.1548	newly	1559.	5981.48	{5857., 6100.}	283.674	0.583911	Reff	0.61568	0.34259	{0.338607, 0.348001}	44.3558	ar	63 222.	101 578.	{101 326., 101 807.}	60.6688
12	R0	{1.70234, 9.34626}	{2.0046, 20.}	{{1.99999, 20.}, {2.00933, 20.}}	{17.7558, 113.989}	inf	5033.	15 421.	{15 372.6, 15 466.7}	206.397	0.486283	newly	3340.	5214.28	{5128., 5286.}	56.1162	0.193419	Reff	0.662312	0.825296	{0.821951, 0.829538}	24.6083	ar	68 213.	86 078.1	{85 786., 86 382.}	26.1901
13	R0	{1.68556, 9.74954}	{2.26857, 20.}	{{2.16006, 20.}, {2.32725, 20.}}	{34.5884, 105.138}	inf	4862.	14 605.2	{14 151.3, 15 429.1}	200.394	0.477475	newly	3072.	4746.36	{4551., 4993.}	54.5039	0.188696	Reff	0.650053	0.600355	{0.58232, 0.623685}	7.64525	ar	68 426.	96 069.1	{94 339., 97 322.}	40.3986
14	R0	{1.59876, 9.78897}	{2.21412, 19.9532}	{{2.18616, 20.}, {2.24694, 20.}}	{38.4896, 103.833}	inf	5531.	15 137.9	{14 907.5, 15 478.2}	173.693	0.437056	newly	3640.	5031.75	{4910., 5142.}	38.2349	0.14055	Reff	0.761314	0.708869	{0.693739, 0.725395}	6.98688	ar	63 553.	95 170.5	{94 489., 96 175.}	49.7498
15	R0	{1.43577, 5.12819}	{2.21106, 20.}	{{2.20043, 20.}, {2.22027, 20.}}	{53.998, 290.001}	inf	2030.	18 320.8	{18 149.3, 18 429.}	802.501	0.95544	newly	2303.	6075.02	{5989., 6179.}	163.787	0.421222	Reff	0.736378	0.726668	{0.719696, 0.734747}	1.33891	ar	53 259.	92 122.3	{91 577., 92 634.}	72.9703
16	R0	{1.58866, 8.73191}	{2.14063, 20.}	{{2.13064, 20.}, {2.14749, 20.}}	{34.7447, 129.045}	inf	5612.	17 627.	{17 513.8, 17 795.2}	214.095	0.497026	newly	4036.	5864.78	{5773., 5969.}	45.3117	0.162234	Reff	0.723984	0.733308	{0.727345, 0.73728}	1.28788	ar	64 349.	93 351.3	{92 717., 93 930.}	45.0704
17	R0	{1.43012, 3.8163}	{2.99039, 20.}	{{2.96475, 20.}, {3., 20.}}	{109.101, 424.068}	inf	1091.	21 320.7	{21 118., 21 516.6}	1854.23	1.29096	newly	1516.	6719.67	{6629., 6826.}	343.25	0.646612	Reff	0.70422	0.420106	{0.407932, 0.431523}	40.3445	ar	53 353.	100 042.	{99 369., 100 557.}	87.51
18	R0	{1.47965, 5.16472}	{2.06886, 14.05}	{{2.04277, 13.8233}, {2.09228, 14.1713}}	{39.8207, 172.037}	inf	1756.	13 300.4	{13 049.6, 13 504.7}	657.425	0.879148	newly	1958.	6004.91	{5877., 6143.}	206.686	0.48661	Reff	0.705823	0.627281	{0.614836, 0.635936}	11.1277	ar	56 086.	96 968.6	{96 348., 97 597.}	72.8927
19	R0	{1.57191, 5.2314}	{2.89647, 20.}	{{2.85871, 20.}, {2.92224, 20.}}	{84.2647, 282.307}	inf	1293.	16 180.9	{16 011.1, 16 508.3}	1151.43	1.09737	newly	1298.	5056.84	{4943., 5181.}	289.587	0.590534	Reff	0.610687	0.363583	{0.355085, 0.375396}	40.4632	ar	63 119.	100 240.	{99 345., 100 652.}	58.8118
20	R0	{1.54435, 5.32911}	{2.31919, 20.}	{{2.31459, 20.}, {2.32319, 20.}}	{50.1728, 275.297}	inf	1762.	19 035.4	{18 975.5, 19 076.}	980.327	1.03355	newly	1849.	6257.7	{6159., 6342.}	238.437	0.529451	Reff	0.632813	0.666691	{0.664099, 0.670558}	5.35352	ar	61 870.	94 481.5	{94 252., 94 726.}	52.7096
21	R0	{1.57932, 4.90031}	{3., 20.}	{{3., 20.}, {3., 20.}}	{89.9552, 308.137}	inf	1650.	19 675.	{19 515.5, 19 872.8}	1092.42	1.07642	newly	1742.	6156.56	{6045., 6245.}	253.419	0.548258	Reff	0.608496	0.371325	{0.368382, 0.374508}	38.9766	ar	63 595.	99 387.1	{98 908., 99 657.}	56.2813
22	R0	{1.47044, 4.07694}	{2.17639, 17.3157}	{{2.12379, 16.6952}, {2.20772, 17.6881}}	{48.0093, 324.722}	inf	1362.	18 390.1	{17 583.8, 18 828.6}	1250.23	1.13021	newly	1880.	7089.38	{6970., 7192.}	277.095	0.576416	Reff	0.691357	0.800953	{0.792817, 0.808771}	15.8523	ar	55 895.	89 233.7	{87 913., 90 109.}	59.6453
23	R0	{1.64997, 3.34546}	{3., 20.}	{{3., 20.}, {3., 20.}}	{81.8215, 497.825}	inf	230.	22 580.3	{22 492.8, 22 676.7}	9717.54	1.992	newly	308.	7091.73	{7012., 7170.}	2202.51	1.36218	Reff	0.558729	0.396776	{0.394789, 0.398675}	28.9861	ar	66 404.	104 294.	{103 787., 104 784.}	57.0597
24	R0	{1.67405, 4.50254}	{3., 20.}	{{3., 20.}, {3., 20.}}	{79.2061, 344.194}	inf	1189.	23 141.6	{23 084.4, 23 193.5}	1846.3	1.28921	newly	1389.	7301.69	{7219., 7381.}	425.68	0.720704	Reff	0.560623	0.423875	{0.421701, 0.426141}	24.3921	ar	68 017.	101 012.	{100 696., 101 303.}	48.5106
25	R0	{1.50663, 3.1286}	{3., 20.}	{{3., 20.}, {3., 20.}}	{99.1199, 539.264}	inf	450.	23 229.6	{23 038.1, 23 581.1}	5062.13	1.71281	newly	698.	7331.19	{7216., 7477.}	950.314	1.02128	Reff	0.634201	0.435731	{0.429066, 0.440156}	31.2944	ar	58 823.	101 752.	{101 213., 102 156.}	72.9798
26	R0	{1.69822, 6.34992}	{2.17715, 17.6698}	{{2.17422, 17.6416}, {2.17877, 17.7102}}	{28.202, 178.269}	inf	2723.	17 297.3	{17 270.4, 17 337.8}	535.231	0.802931	newly	2372.	6450.26	{6359., 6519.}	171.933	0.434444	Reff	0.56493	0.730754	{0.728331, 0.733821}	29.353	ar	69 816.	89 159.8	{88 935., 89 342.}	27.7068
27	R0	{1.55869, 4.92988}	{2.80141, 20.}	{{2.78383, 20.}, {2.81467, 20.}}	{79.7288, 305.689}	inf	1548.	19 738.	{19 661.1, 19 803.2}	1175.06	1.10553	newly	1681.	6249.93	{6164., 6337.}	271.798	0.570281	Reff	0.625643	0.441243	{0.434823, 0.449108}	29.4736	ar	62 344.	98 999.9	{98 707., 99 294.}	58.7962
28	R0	{1.46085, 6.06899}	{1.91526, 13.6101}	{{1.89897, 13.4849}, {1.94169, 13.8307}}	{31.1055, 124.256}	inf	2116.	10 727.7	{10 552.2, 11 155.1}	406.982	0.704889	newly	2108.	5013.79	{4899., 5136.}	137.846	0.376232	Reff	0.722157	0.643116	{0.637038, 0.647353}	10.9451	ar	55 484.	91 770.1	{91 103., 92 962.}	65.3992
29	R0	{1.63017, 6.25164}	{2.94638, 20.}	{{2.89135, 20.}, {2.98119, 20.}}	{80.7404, 219.916}	inf	2357.	16 956.2	{16 640.3, 17 245.7}	619.4	0.85693	newly	2132.	5299.68	{5156., 5423.}	148.578	0.395381	Reff	0.606896	0.36055	{0.347028, 0.377718}	40.5912	ar	66 231.	99 541.	{98 575., 100 333.}	50.2936
30	R0	{1.45173, 5.68165}	{2.1874, 20.}	{{2.17976, 20.}, {2.19347, 20.}}	{50.6757, 252.01}	inf	2119.	15 700.7	{15 610.3, 15 773.5}	640.95	0.869786	newly	2202.	5270.42	{5186., 5347.}	139.347	0.378995	Reff	0.721074	0.781741	{0.777773, 0.785945}	8.41333	ar	55 280.	90 620.	{90 202., 90 961.}	63.9291
31	R0	{1.55808, 2.77316}	{3., 20.}	{{3., 20.}, {3., 20.}}	{92.5447, 621.199}	inf	226.	23 186.8	{23 119.8, 23 245.2}	10 159.7	2.01113	newly	342.	7313.88	{7219., 7409.}	2038.56	1.3301	Reff	0.613478	0.426923	{0.424916, 0.429163}	30.4094	ar	60 940.	100 995.	{100 661., 101 315.}	65.7288
32	R0	{1.56797, 3.90327}	{3., 20.}	{{3., 20.}, {3., 20.}}	{91.3302, 412.391}	inf	914.	22 106.7	{22 065.6, 22 148.6}	2318.68	1.38358	newly	1187.	6958.61	{6852., 7063.}	486.235	0.768044	Reff	0.61673	0.415013	{0.412944, 0.417233}	32.7074	ar	62 228.	99 754.4	{99 523., 100 000.}	60.3047
33	R0	{1.70027, 5.43261}	{3., 20.}	{{3., 20.}, {3., 20.}}	{76.4426, 268.147}	inf	1215.	18 460.2	{18 370.8, 18 541.9}	1419.35	1.18166	newly	1142.	5737.38	{5652., 5810.}	402.398	0.701018	Reff	0.555682	0.326877	{0.325164, 0.328671}	41.1755	ar	68 798.	104 379.	{103 919., 104 790.}	51.7185
34	R0	{1.61668, 6.86192}	{1.9418, 20.}	{{1.93776, 20.}, {1.94542, 20.}}	{20.1102, 191.464}	inf	2934.	14 912.4	{14 814.2, 14 988.4}	408.263	0.706083	newly	2525.	5155.86	{5066., 5233.}	104.192	0.310005	Reff	0.646575	0.957668	{0.955363, 0.960001}	48.1139	ar	64 693.	83 275.9	{82 920., 83 606.}	28.7248
35	R0	{1.55955, 3.45071}	{3., 20.}	{{3., 20.}, {3., 20.}}	{92.3632, 479.591}	inf	514.	24 334.5	{24 232.2, 24 421.4}	4634.35	1.67526	newly	674.	7695.97	{7591., 7812.}	1041.84	1.05758	Reff	0.608474	0.447121	{0.444958, 0.450161}	26.5177	ar	61 783.	103 535.	{103 009., 104 005.}	67.5788
36	R0	{1.65068, 4.91307}	{2.99623, 20.}	{{2.98851, 20.}, {3., 20.}}	{81.5146, 307.077}	inf	1866.	22 436.2	{22 383., 22 565.6}	1102.37	1.08004	newly	2044.	7084.17	{7006., 7164.}	246.584	0.539789	Reff	0.596341	0.434264	{0.432187, 0.436865}	27.1785	ar	66 453.	99 536.	{99 343., 99 705.}	49.7841
37	R0	{1.46131, 3.06714}	{3., 20.}	{{3., 20.}, {3., 20.}}	{105.295, 552.073}	inf	344.	21 072.7	{21 017.3, 21 115.6}	6025.79	1.78716	newly	544.	6609.5	{6529., 6684.}	1114.98	1.08455	Reff	0.646659	0.384177	{0.382654, 0.385856}	40.5905	ar	56 452.	101 980.	{101 676., 102 242.}	80.6486
38	R0	{1.71034, 5.2546}	{3., 20.}	{{3., 20.}, {3., 20.}}	{75.4037, 280.619}	inf	1519.	19 177.3	{19 112.1, 19 220.}	1162.5	1.10123	newly	1497.	5962.69	{5874., 6050.}	298.309	0.600196	Reff	0.547719	0.338247	{0.336194, 0.341204}	38.2445	ar	69 816.	101 894.	{101 374., 102 300.}	45.9472
39	R0	{1.43565, 5.57989}	{1.91669, 13.6821}	{{1.88526, 13.4168}, {1.95317, 14.0925}}	{33.5069, 145.204}	inf	1796.	11 048.	{10 682.3, 11 511.8}	515.142	0.788788	newly	1875.	5154.41	{5026., 5293.}	174.902	0.439097	Reff	0.709096	0.660649	{0.65243, 0.669983}	6.83223	ar	54 407.	88 754.3	{87 901., 89 502.}	63.1304
40	R0	{1.40327, 4.00764}	{2.38087, 15.8958}	{{2.34763, 15.5281}, {2.38908, 15.9574}}	{69.6656, 296.638}	inf	1177.	15 949.8	{15 277.9, 16 175.4}	1255.12	1.13135	newly	1644.	6332.17	{6161., 6463.}	285.168	0.585554	Reff	0.717576	0.551107	{0.53584, 0.562471}	23.1988	ar	51 976.	99 968.9	{99 306., 100 501.}	92.3366
41	R0	{1.63612, 4.26741}	{3., 20.}	{{3., 20.}, {3., 20.}}	{83.3606, 368.668}	inf	805.	20 506.2	{20 448.7, 20 546.7}	2447.36	1.40609	newly	987.	6409.91	{6309., 6499.}	549.434	0.812505	Reff	0.590394	0.360389	{0.358807, 0.362189}	38.9579	ar	65 029.	103 541.	{103 200., 103 896.}	59.2235
42	R0	{1.57426, 6.50271}	{2.20815, 20.}	{{2.20304, 20.}, {2.21204, 20.}}	{40.2656, 207.564}	inf	2814.	18 504.3	{18 412.5, 18 576.6}	557.582	0.817947	newly	2604.	6119.92	{6010., 6207.}	135.02	0.371074	Reff	0.666321	0.711314	{0.707811, 0.715655}	6.75248	ar	62 522.	94 810.9	{94 336., 95 190.}	51.6441
43	R0	{1.37818, 5.37636}	{2.25491, 20.}	{{2.23589, 20.}, {2.26576, 20.}}	{63.6153, 271.999}	inf	1811.	16 293.1	{16 127.1, 16 464.6}	799.672	0.954069	newly	2047.	5404.79	{5293., 5501.}	164.035	0.421615	Reff	0.756841	0.724499	{0.71125, 0.739749}	4.27327	ar	50 098.	97 004.2	{96 458., 97 686.}	93.629
44	R0	{1.66606, 4.92641}	{3., 20.}	{{3., 20.}, {3., 20.}}	{80.0655, 305.975}	inf	1739.	22 529.	{22 466., 22 570.4}	1195.52	1.11244	newly	1807.	7099.42	{6997., 7190.}	292.884	0.594241	Reff	0.583371	0.420332	{0.418076, 0.422698}	27.9478	ar	67 066.	100 104.	{99 854., 100 329.}	49.2622
45	R0	{1.623, 5.9449}	{2.86512, 20.}	{{2.80837, 20.}, {2.90879, 20.}}	{76.5323, 236.423}	inf	2642.	19 857.5	{19 653.3, 20 091.2}	651.608	0.875975	newly	2549.	6276.53	{6156., 6407.}	146.235	0.391301	Reff	0.641799	0.436687	{0.422574, 0.454246}	31.959	ar	64 554.	99 113.7	{97 613., 99 878.}	53.5361
46	R0	{1.65235, 5.51234}	{2.14378, 17.0112}	{{2.14097, 16.9925}, {2.1464, 17.049}}	{29.7413, 208.603}	inf	2356.	17 681.2	{17 645.2, 17 710.4}	650.475	0.875336	newly	2372.	6947.68	{6851., 7032.}	192.904	0.466705	Reff	0.608825	0.819879	{0.81751, 0.822247}	34.6659	ar	66 509.	87 454.4	{87 168., 87 697.}	31.4926
47	R0	{1.62138, 8.82208}	{2.16019, 19.2061}	{{2.03488, 17.6308}, {2.25931, 20.}}	{33.2316, 117.705}	inf	4208.	14 224.1	{12 691.2, 15 255.4}	238.025	0.527649	newly	2927.	4798.46	{4627., 4990.}	63.9378	0.214488	Reff	0.666095	0.604725	{0.572494, 0.629209}	9.2134	ar	65 598.	91 492.	{88 204., 93 660.}	39.4737
48	R0	{1.64073, 6.69261}	{2.83342, 19.4451}	{{2.518, 17.3783}, {2.96938, 20.}}	{72.6928, 190.546}	inf	3295.	18 193.3	{15 832.9, 19 086.2}	452.149	0.741108	newly	2847.	5897.5	{5760., 6030.}	107.148	0.316208	Reff	0.626628	0.42594	{0.392096, 0.49745}	32.0266	ar	66 438.	99 034.	{96 299., 100 687.}	49.0623
49	R0	{1.65556, 3.796}	{3., 20.}	{{3., 20.}, {3., 20.}}	{81.2076, 426.87}	inf	539.	25 206.8	{25 100.8, 25 295.1}	4576.58	1.66993	newly	685.	7970.9	{7871., 8054.}	1063.64	1.0658	Reff	0.564513	0.454692	{0.452373, 0.456673}	19.454	ar	66 575.	103 378.	{102 941., 103 870.}	55.2798
50	R0	{1.56828, 3.65713}	{3., 20.}	{{3., 20.}, {3., 20.}}	{91.2924, 446.877}	inf	506.	20 944.9	{20 868.3, 21 009.9}	4039.3	1.61693	newly	685.	6545.88	{6436., 6628.}	855.603	0.980249	Reff	0.595868	0.364224	{0.362118, 0.366156}	38.875	ar	62 761.	104 159.	{103 708., 104 564.}	65.9612
